# Diagnostic test interpretation and referral delay in patients with interstitial lung disease

**DOI:** 10.1186/s12931-019-1228-2

**Published:** 2019-11-12

**Authors:** David Pritchard, Ayodeji Adegunsoye, Elyse Lafond, Janelle Vu Pugashetti, Ryan DiGeronimo, Noelle Boctor, Nandini Sarma, Isabella Pan, Mary Strek, Michael Kadoch, Jonathan H. Chung, Justin M. Oldham

**Affiliations:** 1 Department of Internal Medicine, University of California at Davis, Davis, USA; 2 Department of Medicine; Section of Pulmonary, Critical Care Medicine, University of Chicago, Chicago, USA; 3 Division of Pulmonary and Critical Care Medicine, Weill Cornell Medicine, New York, USA; 4 Department of Internal Medicine; Division of Pulmonary, Critical Care and Sleep Medicine, University of California at Davis, Davis, USA; 5 Department of Radiology, University of California, Davis, USA; 6 Department of Radiology, University of Chicago, Chicago, USA; 7 Department of Veterans Affairs Northern California, 4150 V Street Suite 3400, Sacramento, CA 95817 USA

**Keywords:** Interstitial lung disease, Diagnostic delay, Idiopathic pulmonary fibrosis, Pulmonary function test, Computed tomography

## Abstract

**Background:**

Diagnostic delays are common in patients with interstitial lung disease (ILD). A substantial percentage of patients experience a diagnostic delay in the primary care setting, but the factors underpinning this observation remain unclear. In this multi-center investigation, we assessed ILD reporting on diagnostic test interpretation and its association with subsequent pulmonology referral by a primary care physician (PCP).

**Methods:**

A retrospective cohort analysis of patients referred to the ILD programs at UC-Davis and University of Chicago by a PCP within each institution was performed. Computed tomography (CT) of the chest and abdomen and pulmonary function test (PFT) were reviewed to identify the date ILD features were first present and determine the time from diagnostic test to pulmonology referral. The association between ILD reporting on diagnostic test interpretation and pulmonology referral was assessed, as was the association between years of diagnostic delay and changes in fibrotic features on longitudinal chest CT.

**Results:**

One hundred and forty-six patients were included in the final analysis. Prior to pulmonology referral, 66% (*n* = 97) of patients underwent chest CT, 15% (*n* = 21) underwent PFT and 15% (n = 21) underwent abdominal CT. ILD features were reported on 84, 62 and 33% of chest CT, PFT and abdominal CT interpretations, respectively. ILD reporting was associated with shorter time to pulmonology referral when undergoing chest CT (1.3 vs 15.1 months, respectively; *p* = 0.02), but not PFT or abdominal CT. ILD reporting was associated with increased likelihood of pulmonology referral within 6 months of diagnostic test when undergoing chest CT (rate ratio 2.17, 95% CI 1.03–4.56; *p* = 0.04), but not PFT or abdominal CT. Each year of diagnostic delay was associated with a 1.8% increase in percent fibrosis on chest CT. Patients with documented dyspnea had shorter time to chest CT acquisition and pulmonology referral than patients with documented cough and lung crackles.

**Conclusions:**

Determinants of ILD diagnostic delays in the primary care setting include underreporting of ILD features on diagnostic testing and prolonged time to pulmonology referral even when ILD is reported. Interventions to modulate these factors may reduce ILD diagnostic delays in the primary care setting.

## Introduction

Interstitial lung disease (ILD) is made up of a heterogeneous group of diffuse parenchymal lung disorders that often result in pulmonary fibrosis. ILD results in a high burden of disease, leading to increased healthcare utilization [1, 2] and reduced survival [3]. Despite these observations, diagnostic delays remain common. A majority of patients report waiting more than 1 year from the time of symptom onset to ILD diagnosis and often undergo multiple physician evaluations before arriving at the correct diagnosis [4]. Even with recognition of this problem, little headway has been made over the last decade [5, 6].

A recent investigation by Hoyer and colleagues showed that ILD diagnostic delays occur in several settings, with the primary care setting playing a prominent role [7]. Factors leading to ILD diagnostic delays remain unclear, but may stem from unrecognized or underappreciated features of ILD on diagnostic testing. ILD is best characterized by computed tomography (CT) of the chest and can range from subtle, sub-clinical reticular opacities to parenchymal destruction characterized by traction bronchiectasis and honeycombing [8–10]. ILD can also be detected by abdominal CT, as lung bases are commonly captured by this modality. While CT confirms the presence of ILD, physiologic features of ILD can be detected on pulmonary function testing (PFT), including reductions in total lung capacity (TLC), forced vital capacity (FVC) and diffusion capacity of the lung for carbon monoxide (DLCO) [11].

Studies to date characterizing diagnostic delays in patients with ILD have largely relied on patient questionnaires and insurance claims data [4–7]. While informative, these studies do not assess physician-level factors that may influence such delays. We showed previously that underreporting of interstitial lung abnormalities on lung cancer screening CT was associated with delays in pulmonology referral [12], which suggests a similar phenomenon may occur with diagnostic tests performed prior to ILD diagnosis. In this multi-center investigation, we reviewed all prior chest CTs, abdominal CTs and PFTs performed in patients referred to the ILD programs at UC-Davis and University of Chicago to determine the time from first diagnostic test showing features of ILD to pulmonology referral. We hypothesized that ILD reporting on diagnostic test interpretation would lead to reduced time to pulmonology referral. We then assessed how diagnostic delays influenced progression of pulmonary fibrosis on longitudinal chest CT and characterized time to chest CT and pulmonology referral following documentation of ILD clinical features, including cough, dyspnea and lung crackles.

## Methods

This retrospective cohort investigation was performed at the University of California at Davis (UC-Davis) and the University of Chicago (UChicago). The electronic medical record was reviewed for all patients with an institutional PCP who consented to participate in the ILD registry at each center (UC-Davis IRB protocol #928979 and UChicago IRB protocol #13–1180). Patients with prevalent ILD diagnosed by an outside pulmonologist prior to ILD program referral were excluded, as were patients with missing clinical data or CT images.

Relevant data extracted from the electronic medical record included demographics (age, sex, race, smoking history), ILD signs and symptoms, ILD diagnosis, date of pulmonology referral, date and interpretation of all chest CT, abdominal CT and PFT. ILD features were considered reported on CT when there was mention of “reticulation,” “reticular opacities” “honeycombing” “traction bronchiectasis” “traction bronchiolectasis” “ground glass opacity” “interstitial changes” “fibrosis” and “fibrotic changes” on the radiology report. ILD was considered reported on PFT when ILD was mentioned in the PFT interpretation, including when listed as part of a differential diagnosis.

All chest and abdominal CTs performed prior to pulmonology referral were reviewed by a thoracic radiologist (MK at UC-Davis and JHC at UChicago) to ascertain the date of first CT with evidence of ILD. For chest CTs, ILD was defined as bilateral, non-dependent reticular opacities affecting > 5% of the lung. For abdominal CT, ILD was defined as bilateral, non-dependent reticular opacities at the lung bases. All PFTs performed prior to pulmonology referral were reviewed by JMO and AA to ascertain the date of first PFT with ILD features, defined as TLC, FVC or DLCO was < 80% predicted. Paired chest CTs performed ≥6 months prior to pulmonology referral and within 6 months of pulmonology evaluation at UC-Davis were then scored to determine the longitudinal change in fibrosis extent, defined as the sum of whole lung percent reticular opacities and honeycombing.

To explore the association between ILD clinical feature onset and subsequent ILD work-up, the time from documentation of cough, dyspnea and lung crackles to chest CT and pulmonology referral was assessed. ILD clinical features were considered present when PCP documentation of was sustained prior to chest CT and pulmonology referral. These data could only be assessed in the UC-Davis cohort, as a transition from paper to electronic documentation during the study period precluded reliable acquisition of PCP records in the UChicago cohort.

### Statistical analysis

Continuous variables are presented as means ± standard deviation or as medians with interquartile range based on variable distribution. Time from diagnostic test to pulmonology referral is compared between ILD reporting groups using a generalized Wilcoxon test weighted for early events and displayed using the Kaplan Meier estimator. The association between diagnostic testing interpretation and pulmonology referral within 6 months of testing was assessed using Poisson regression with robust error variance [13]. Longitudinal change in fibrotic features on chest CT associated with diagnostic delay was assessed using a maximum likelihood linear mixed effects model with time between CTs aligned to 1-year intervals. Age, sex, race, ILD subtype and smoking history were included as fixed effects variables to adjust for potential confounders of fibrotic change over time. An autoregressive correlation structure and random slope term were chosen for longitudinal modeling based on exploratory analysis using restricted maximum likelihood testing. Longitudinal change in CT measures are displayed graphically using locally weighted scatterplot smoothing. All analyses were performed using Stata (Release 16; StataCorp, College Station, TX, USA). Statistical significance was set at *p* < 0.05.

## Results

**Fig. 1:**
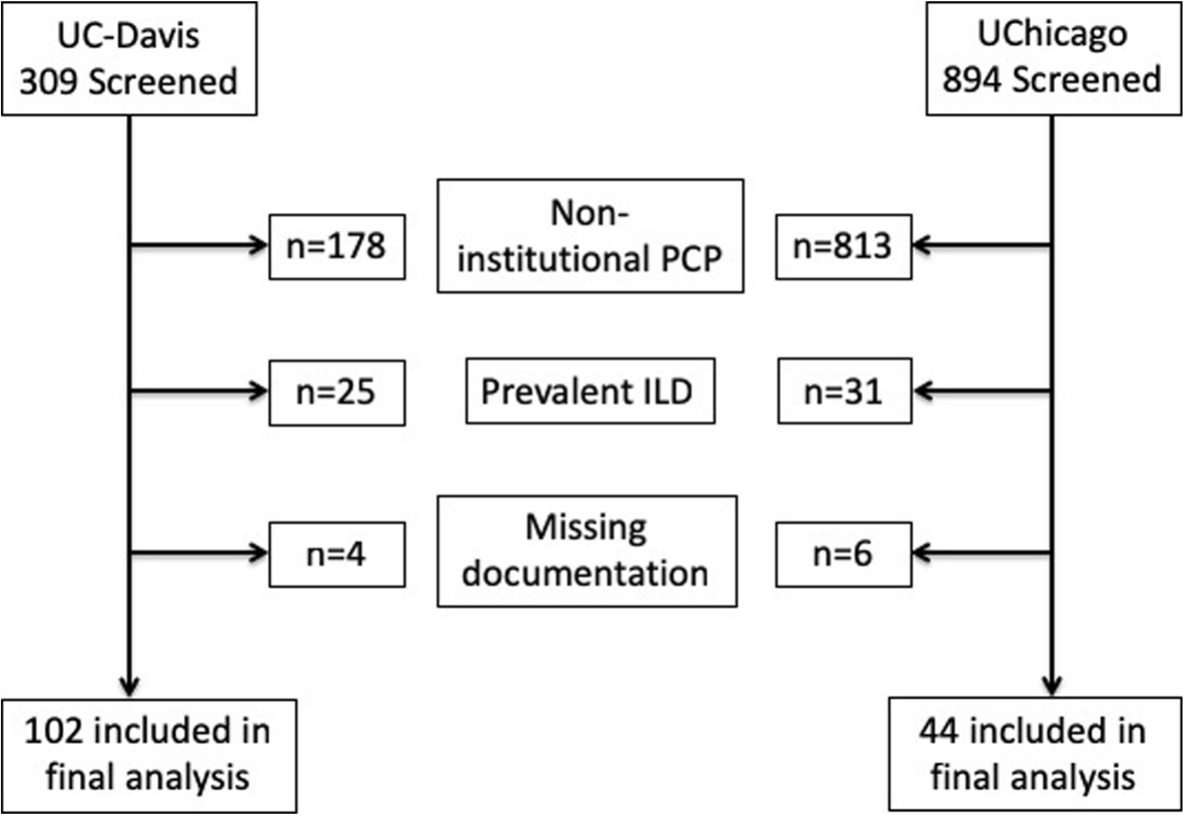
Consort/STROBE diagram

Of 309 patients screened at UC-Davis, 207 were excluded, including 178 without an institutional PCP. Of 894 screened at UChicago, 850 were excluded, including 813 without an institutional PCP. One hundred and two patients from UC-Davis and 44 from UChicago were included in the final analysis (Fig. 1). Baseline characteristics for each cohort are shown in Table 1. Those with ILD from UChicago were younger and had a higher percentage of females and African Americans than those from UC-Davis. Smoking history and reason for pulmonology referral were similar between groups. ILD diagnosis varied between centers, with unclassifiable pulmonary fibrosis being most common at UCD-Davis and connective tissue disease-associated ILD being most common at UChicago. Idiopathic pulmonary fibrosis (IPF) accounted for approximately 25% of both cohorts. Patients at UC-Davis had better overall lung function than patients in the UChicago cohort.

**Table 1:** Baseline Characteristics upon ILD Program Evaluation

Variable	UCD Cohort (*n* = 102)	UChicago Cohort (*n* = 44)
Age, mean (SD)	72.5 (9.5)	59.8 (14.1)
Male, n (%)	64 (62.8)	17 (38.6)
Race, n (%)		
White	87 (85.3)	9 (20.5)
African American	3 (2.9)	34 (77.3)
Hispanic	5 (4.9)	1 (2.3)
Asian	7 (6.9)	0 (0)
Smoking History		
Never, n (%)	38 (37.3)	17 (38.6)
Former or current, n (%)	64 (62.8)	27 (61.4)
Pack-years if former/current, median (IQR)	20 (10–42.5)	22 (15–40)
Pulmonary Referral Reason		
Abnormal imaging/ILD, n (%)	63 (61.8)	35 (79.6)
Cough and/or dyspnea, n (%)	19 (18.6)	7 (15.9)
Abnormal PFT	6 (5.9)	1 (2.3)
Other, n (%)	14 (13.7)	1 (2.3)
ILD Diagnosis, n (%)		
Idiopathic pulmonary fibrosis	28 (27.5)	12 (27.3)
CTD-associated ILD	20 (19.6)	22 (50)
Chronic HP	10 (9.8)	2 (18.2)
Unclassifiable Fibrosis	34 (33.3)	8 (18.2)
Other ILD	10 (9.8)	0 (0)
Pulmonary Function		
Total lung capacity (% predicted), mean (SD)	79 (17)	72 (18)
Forced vital capacity (% predicted), mean (SD)	83 (19)	66 (18)
Diffusion capacity (% predicted), mean (SD)	59 (17)	54 (20)

### Chest computed tomography

**Fig. 2:**
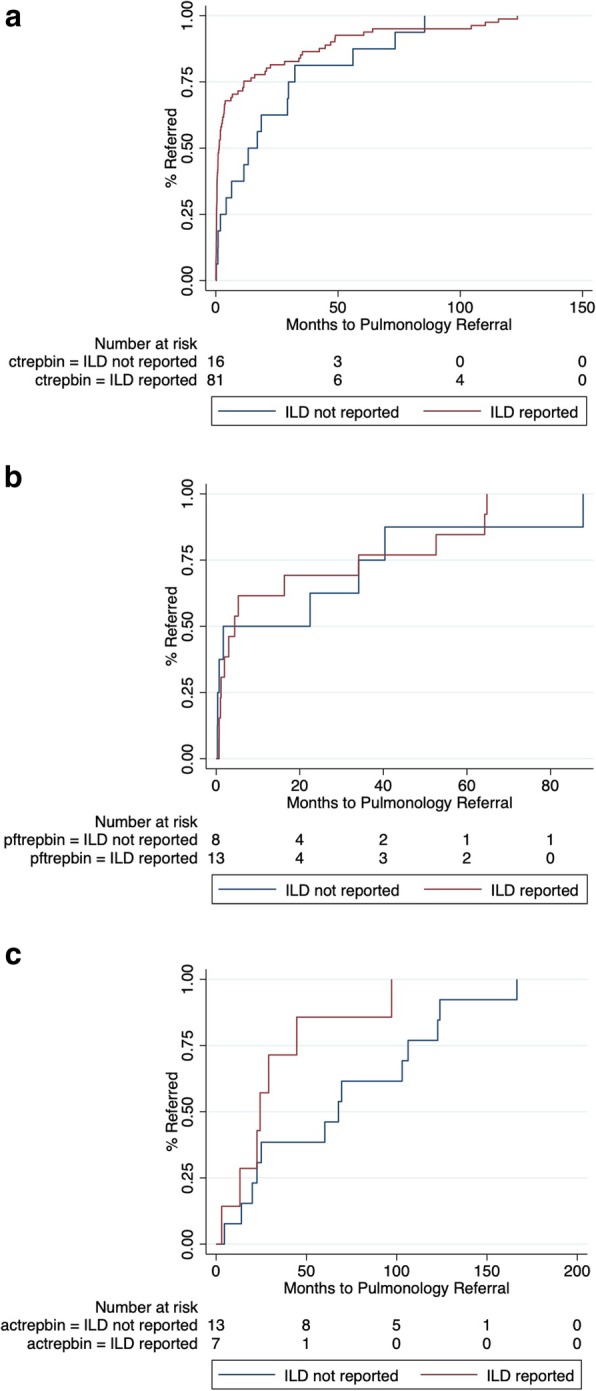
Time to pulmonology referral stratified by ILD reporting in those undergoing chest computed tomography (**a**), pulmonary function testing (**b**) and abdominal computed tomography (**c**)

A chest CT with features of ILD was obtained in 66% (*n* = 97) of patients prior to pulmonology referral, with ILD reported on chest CT interpretation in 84% (*n* = 81/97) of cases (Table 2). The median time to pulmonology referral was 1.3 months (IQR 0.3–11.5 months) in those for whom ILD was reported and 15.1 months (IQR 3.0–31.0 months) in those for whom ILD was not reported (*p* = 0.02) (Fig. 2a). A pulmonology referral was placed within 6 months of chest CT in 68% (*n* = 55/81) of patients for whom ILD was reported and 31% (n = 5/16) for whom ILD was not reported (Table 3). Reporting of ILD on chest CT interpretation was associated with a two-fold increase in likelihood of pulmonology referral within 6 months of chest CT (rate ratio (RR) 2.17; 95% CI 1.03–4.56; *p* = 0.04). Despite these findings, the time from chest CT to pulmonology referral was > 1 year for roughly 25% of patients for whom ILD was reported on chest CT. A similar time to pulmonology referral was observed between IPF and non-IPF cohorts (20.4 vs 13.9 months, respectively; *p* = 0.41).

**Table 2:** Time to chest CT and pulmonology referral after diagnostic testing in patients with ILD

Variable	ILD reported	ILD not reported	*p*-value
Chest CT			
Chest CT with ILD features^a^ obtained prior to pulmonology referral, n (%)	81/97 (83.5)	16/97 (16.5)	
Months from chest CT to pulmonology referral, median (IQR)	1.3 (0.3–11.5)	15.1 (3.0–31.0)	0.02
Pulmonary Function Testing			
PFT with ILD features^b^ obtained by PCP prior to chest CT and pulmonology referral, n (%)	13/21 (61.9)	8/21 (38.1)	
Months from PFT to chest CT when ILD reported by pulmonologist, median (IQR)	3.7 (0.8–14.8)	13.1 (1.6–27.8)	0.2
Months from PFT to pulmonology referral when ILD reported, median (IQR)	4.4 (1.2–34.1)	12.1 (0.5–37.2)	0.74
Abdominal CT			
Abdominal CT with ILD features^c^ obtained prior to chest CT and pulmonology referral, n (%)	7/21 (33.3)	14/21 (66.7)	
Months from abdominal CT to chest CT when ILD not reported by radiologist, median (IQR)	12.3 (2.3–28.2)	21.4 (17.9–70.7)	0.1
Months from abdominal CT to pulmonology referral when ILD reported by radiologist, median (IQR)	24.4 (13.2–44.6)	63.9 (20.0–106.3)	0.11

^a^Bilateral, non-dependent reticular opacities affecting > 5% of lung area

^b^TLC or FVC or DLCO < 80% predicted

^c^Bilateral, non-dependent reticular opacities at lung bases

**Table 3:** Association between 6-month pulmonology referral and diagnostic testing

	ILD Features Reported on Diagnostic Testing Interpretation					
	(+)	(−)	(+)	(−)	(+)	(−)
	Chest CT		Pulmonary Function Testing		Abdominal CT	
n with referral time < 6 months/total (%)	55/81 (67.9)	5/16 (31.3)	8/13 (61.5)	4/8 (50)	1/7 (14.3)	2/14 (14.3)
Rate ratio	2.17	Ref	1.23	Ref	1	Ref
*p*-value	0.04	Ref	0.62	Ref	1	Ref
95% Confidence Interval	1.03–4.56	Ref	0.54–2.78	Ref	0.11–9.23	Ref

### Pulmonary function testing

A PFT with features of ILD was obtained in 14% (*n* = 21) of patients prior to chest CT and pulmonology referral, with ILD reported on PFT interpretation in 62% (*n* = 13/21) of cases (Table 2). The median time from PFT to chest CT was 3.7 months (IQR 0.8–14.8 months) in those for whom ILD was reported on PFT and 13.1 months (IQR 1.6–27.8 months) in those for whom ILD was not reported (*p* = 0.2) on PFT. The median time to pulmonology referral was 4.4 months (IQR 1.2–34.1 months) in those for whom ILD was reported on PFT and 12.1 months (IQR 0.5–37.2 months) in those for whom ILD was not reported on PFT (*p* = 0.74) (Fig. 2b). A pulmonology referral was placed within 6 months of PFT in 62% (*n* = 8/13) of patients for whom ILD was reported and 50% (*n* = 4/8) of those for whom ILD was not reported (Table 3). Reporting of ILD on chest PFT interpretation was not associated with differential likelihood of pulmonology referral within 6 months of PFT (RR 1.23; 95% CI 0.54–2.78; *p* = 0.62).

### Abdominal computed tomography

An abdominal CT with features of ILD was obtained in 14% (*n* = 21) of patients prior to chest CT and pulmonology referral, with ILD reported on PFT interpretation in 33% (*n* = 7/21) of cases (Table 2). The median time from abdominal CT to chest CT was 12.3 months (IQR 2.3–28.2 months) in those for whom ILD was reported on abdominal CT interpretation and 21.4 months (IQR 17.9–70.7 months) in those for whom ILD was not reported (*p* = 0.1). The median time to pulmonology referral was 24.4 months (IQR 13.2–44.6 months) in those for whom ILD was reported of abdominal CT interpretation and 63.9 months (IQR 20.0–106.3 months) in those for whom ILD was not reported (*p* = 0.11) (Fig. 2c). A pulmonology referral was placed within 6 months of abdominal CT in 14% (*n* = 1/7) of patients for whom ILD was reported on abdominal CT and 14% (*n* = 2/14) for whom ILD was not reported. Reporting of ILD on chest PFT interpretation was not associated with differential likelihood of pulmonology referral within 6 months of abdominal CT (RR 1; 95% CI 0.11–9.23; *p* = 1.0) (Table 3).

### Diagnostic delays and progression of pulmonary fibrosis

**Fig. 3:**
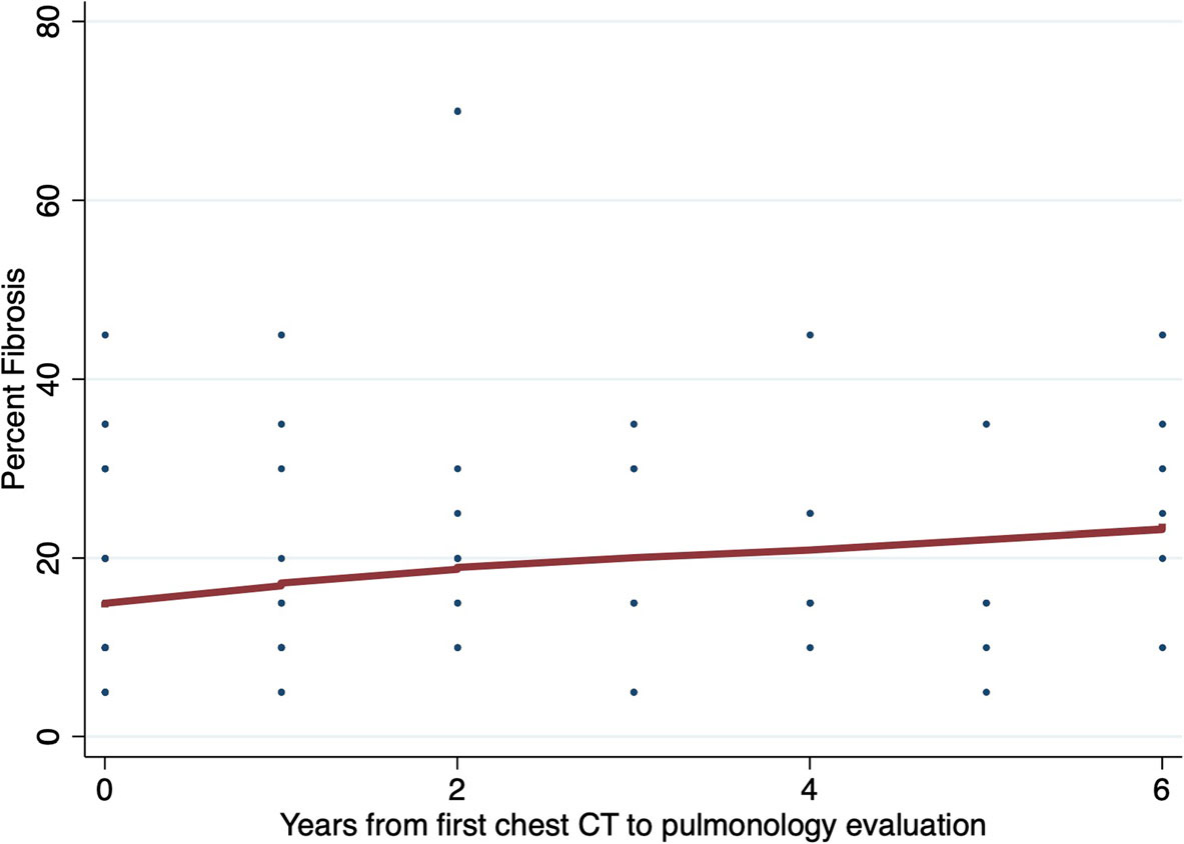
Yearly change in percent fibrosis associated with ILD referral delay

Fifty-one patients had paired chest CT performed prior to and at the time of ILD program evaluation, including 32 from UC-Davis and 19 from UChicago. The median time between chest CTs was 29 months (IQR 10–46). With a baseline percent fibrosis of 14.6%, each year delay prior to ILD program evaluation was associated with a 1.8% (95% CI 1.08–2.57, *p* < 0.001) increase in fibrosis extent (Fig. 3). This was most pronounced among patients with IPF, with each year delay prior to ILD program evaluation associated with a 2.94% (95% CI 0.86–5.03%; *p* = 0.006) increase in fibrosis extent. Non-IPF ILDs had similar yearly increases in fibrosis extent, with CTD-ILD demonstrating an annual increase of 1.22% (95% CI -0.17-2.62; *p* = .09) and combined other ILDs demonstrating an annual increase of 1.33% (95% CI 0.76–1.90; *p* < 0.001).

### Relationship between ILD clinical feature onset, chest CT acquisition and pulmonology referral

At least one clinical feature of ILD was documented by a PCP in 87% of cases (*n* = 89) (Table 4), including 64% (*n* = 66) with cough, 53% (*n* = 54) with dyspnea and 35% (*n* = 36) with lung crackles. The median time from cough documentation to pulmonology referral was 13.2 months (IQR 1.8–58.9 months), with 41% (*n* = 27/66) of patients receiving a pulmonology referral within 6 months of cough onset. Among patients who underwent chest CT prior to pulmonology referral, the median time from documented cough to chest CT was 29.1 months (IQR 0.7–63.0), with 29% (*n* = 13) of patients undergoing chest CT within 6 months of documented cough onset. The median time from dyspnea documentation to pulmonology referral was 1.6 months (IQR 0.7–5.5 months), with 69% (*n* = 37/54) of patients receiving a pulmonology referral within 6 months of dyspnea onset. Among patients who underwent chest CT prior to pulmonology referral, the median time from documented dyspnea to chest CT was 1.2 months (IQR 0.4–4.1), with 41% (*n* = 14/34) of patients undergoing chest CT within 6 months of documented dyspnea onset. The median time from lung crackles documentation to pulmonology referral was 16.3 months (IQR 2.3–37.1 months), with 36% (*n* = 13/36) of patients receiving a pulmonology referral within 6 months of lung crackles onset. Among patients who underwent chest CT prior to pulmonology referral, the median time from documented lung crackles to chest CT was 10.8 months (IQR 0.8–37.3), with 23% (*n* = 7/30) of patients undergoing chest CT within 6 months of documented lung crackles onset.

**Table 4:** Time from documented ILD clinical feature to chest CT and pulmonology referral

Variable	UCD Cohort (*n* = 102)
Any ILD clinical feature documented, n (%)	89 (87.3)
Chronic cough, n (%)	66 (64.7)
Dyspnea, n (%)	54 (52.9)
Lung crackles, n (%)	36 (35.3)
Months from ILD clinical feature to chest CT	
Chronic cough, median (IQR)	29.1 (0.7–63)
Dyspnea, median (IQR)	1.2 (0.4–4.1)
Lung crackles, median (IQR)	10.8 (0.8–37.3)
Months from ILD clinical feature pulmonology referral	
Chronic cough, median (IQR)	13.2 (1.8–58.9)
Dyspnea, median (IQR)	1.6 (0.7–5.5)
Lung crackles, median (IQR)	16.3 (2.3–37.1)

## Discussion

In this investigation we assessed physician-level factors influencing diagnostic delays in patients with ILD across two academic institutions. We found that ILD reporting was highly variable across key diagnostic tests, including 84% reporting on chest CT, 62% on PFT and only 33% on abdominal CT. While ILD reporting was associated with reduced time to pulmonology referral across each diagnostic testing modality, only chest CT reached statistical significance. We also showed that each year of diagnostic delay was associated with a 1.8% increase in fibrosis extent on longitudinal chest CT and that documented dyspnea was associated with more prompt chest CT acquisition and pulmonology referral than documented cough or lung crackles. This study is among the first to assess physician-level factors contributing to ILD diagnostic delays and sheds important light on potential targets for intervention.

ILD reporting was most strongly associated with prompt pulmonology referral in patients undergoing chest CT. We found that ILD was reported on 84% of chest CTs and that this reporting increased by two-fold the likelihood of pulmonology referral within 6 months of chest CT. However, we also found that nearly 25% of patients for whom ILD was reported on chest CT still waited more than year before receiving a pulmonology referral. These data support our previous findings, in which underreporting of interstitial lung abnormalities was common in patients undergoing chest CT for lung cancer screening and was associated with delays in pulmonology referral [12]. Adoption of a standardized chest CT reporting template for ILD, as was recently proposed by Berkowitz and colleagues [14], may improve ILD reporting by providing a standardized framework for describing common ILD features. While systematic screening of all chest CT performed at an institution would likely improve ILD reporting, a more practical approach given the relatively high prevalence of ILD reporting may be systematic screening of chest CT reports followed by a recommendation for pulmonology referral when ILD features are reported.

While a large percentage of patients underwent chest CT prior to pulmonology referral in this study, only 14% of patients underwent PFT prior to referral and of those patients, only 62% of those had ILD reported on the PFT interpretation despite the presence of ILD features. ILD reporting was associated with a shorter time to chest CT and pulmonology referral, but neither of these associations reached statistical significance. We also found that ILD reporting on PFT interpretation was not associated with differential likelihood of pulmonology referral within 6 months of PFT. ILD often results in characteristics changes on PFT, including reductions in TLC, FVC and/or DLCO [11]. However, isolated or composite reductions in these metrics can be observed in numerous unrelated disease states [15–19], which may explain why mention of ILD was less strongly associated with pulmonology referral in these patients. Systematic screening of PFTs followed by a recommendation for chest CT or pulmonology referral when ILD features are present may facilitate a reduction in diagnostic delays observed in these patients. The PFT metrics that most reliably discriminate ILD among all-comers remains unclear, but Suliman and colleagues showed that specific composite measures had a sensitivity of > 70% for detecting ILD in patients with scleroderma [15].

While improved reporting of ILD may facilitate a reduced time to pulmonology referral in patients undergoing chest CT, it is not clear from our data that improved ILD reporting would result in a similar outcome in patients undergoing abdominal CT. We found that ILD was reported in only 33% of abdominal CT interpretations. While this reporting was associated with a reduced time to pulmonology referral, this association was not statistically significant and the median time to pulmonology referral was greater than 2 years. These findings suggest that efforts are needed to both improve ILD reporting on abdominal CT and to facilitate pulmonology referral once ILD is reported. A review of lung windows by a thoracic radiologist for these studies could help address the ILD reporting component, while systematic screening of abdominal CT reports in the setting of improved reporting could help address the referral component.

The non-specific nature of ILD symptoms likely contributes to the diagnostic delays observed in this population. Chronic cough and dyspnea are the predominate symptoms in patients with ILD [6], but are commonly observed with other lung diseases and non-pulmonary etiologies. Our data suggest that most patients with documented dyspnea receive prompt chest CT and pulmonology referral, but that substantial delays occur in patients with documented chronic cough and lung crackles. We found that the median time to pulmonology referral of > 1 year following documentation of cough and lung. While not all patients with chronic cough warrant a chest CT, a recommendation for this diagnostic modality for patients who fail to improve with standard treatments such as bronchodilators, anti-reflux therapy and sinus hygiene should be considered. Lung crackles should prompt a more expeditious work-up. While lung crackles can sometimes be auscultated in the dependent lung portions of healthy individuals [20], they should be considered pathologic when failing to resolve over several breath cycles. The presence of pathologic lung crackles are strongly predictive of pulmonary fibrosis [21], and have accordingly been proposed as a screening tool for ILD [22].

While our study focused on factors influencing diagnostic delays within two academic institutions, Hoyer and colleagues recently provided a more global assessment of diagnostic delays in patients with ILD using national health data in Denmark [7]. Similar primary care-related delays were observed, but significant delays related to patient factors and within the community pulmonologist setting were also identified. Notably, small but measurable delays were also observed prior to ILD specialty center evaluation and while undergoing the ILD work-up within specialty centers. While our study provides some insight into factors influencing diagnostic delays in the primary care setting, the work of Hoyer and colleagues suggests that other avenues for intervention exist.

Our study has several limitations. First, given the retrospective nature of the study, our study allowed only for assessment of association and not causation. Next, while this was a multi-center investigation, the sample size was small and likely resulted in an underpowered study for detection of association between ILD reporting and time to pulmonology referral in those undergoing PFT and abdominal CT. Next, heterogeneity existed between cohorts with regard to baseline characteristics and individual outcome measures, suggesting that center-specific considerations may be needed when formulating strategies to reduce ILD diagnostic delays. Finally, because of our study design, we could only assess factors influencing diagnostic delays within our health systems, both of which are academic medical centers with a large external referral population relative to internal referrals. Whether these results are generalizable to cohorts drawn from community centers remains unclear; however, the consistencies of our findings with those of others who have included patients from community centers do suggest some degree of generalizability [5–7].

## Conclusion

The high prevalence of diagnostic delays in patients with ILD was first reported over a decade ago and recent studies suggest that little has changed since that time. We and others have begun to identify specific factors that influence diagnostic delays in patients with ILD, providing potential strategies to positively impact this unfortunate phenomenon. Additional research is needed to identify cost effective strategies to reduce diagnostic delays, including physician education and systematic screening programs.

## Data Availability

The datasets used and/or analyzed during the current study are available from the corresponding author on reasonable request.

## Abbreviations

CT: Computed tomography
DLCO: Diffusion capacity of the lung for carbon monoxide
FVC: Forced vital capacity
ILD: Interstitial lung disease
PFT: Pulmonary function testing
TLC: Total lung capacity
